# Possibility of Worsening Flow Diversion Effect Due to Morphological Changes of a Stented Artery With Multiple Overlapping Stents for Partially Thrombosed Vertebral Artery Aneurysms

**DOI:** 10.3389/fneur.2020.611124

**Published:** 2020-12-15

**Authors:** Tomoaki Suzuki, Hitoshi Hasegawa, Kazuhiro Ando, Kouhei Shibuya, Haruhiko Takahashi, Shoji Saito, Makoto Oishi, Yukihiko Fujii

**Affiliations:** Department of Neurosurgery, Brain Research Institute, Niigata University, Niigata, Japan

**Keywords:** overlapping stents, posterior circulation, vertebral artery aneurysm, computational fluid dynamics, morphological change

## Abstract

**Background:** Morphological changes of a stented artery can cause a flow diversion effect to reduce intra-aneurysmal flow; however, there is a potential for the negative effect of increased intra-aneurysmal flow. We present cases with multiple overlapping stents for a partially thrombosed vertebral artery aneurysm and characterize the hemodynamic properties of a recurrent case by focusing on the morphological changes of the stented artery.

**Methods:** Between October 2017 and April 2019, four consecutive cases of symptomatic unruptured large and giant partially thrombosed vertebral artery aneurysms were treated with multiple overlapping low-profile visualized intraluminal support stents and no coils. Both angiographic and clinical outcomes were assessed. Computational fluid dynamics analysis was performed to clarify hemodynamic features. The degree of pressure elevation was calculated as the pressure difference (Pd). Wall shear stress (WSS) was also calculated.

**Results:** In three of the four cases, successful flow reduction was achieved with no morphological change of the stented arteries. The patients' symptoms were gradually improved. The remaining case required additional stents after the initial treatment. In the recurrent case, Pd was noticeably elevated at the aneurysm neck after treatment, and WSS was generally increased in the area due to altered blood flow into the aneurysm dome caused by morphological changes of the stented artery.

**Conclusion:** Overlapping stents can be used for the treatment of large and giant thrombosed vertebral artery aneurysms with flow diversion effect; however, morphological changes of the stented artery requires careful attention as it may lead to an increase in the intra-aneurysmal flow, causing negative outcomes.

## Introduction

In endovascular surgery using neck-bridge stents and flow diverters (FDs), the flow diversion effect is an important factor in occlusion of cerebral aneurysms and is caused by metal coverage at the aneurysm orifice as well as straightening of the stented parent artery (1–4). Reduction of intra-aneurysmal flow often contributes positively to the treatment of cerebral aneurysms. However, unpredictable flow changes are possible with this treatment (5–7). Previous computational fluid dynamics (CFD) studies have shown that multiple low-profile visualized intraluminal support (LVIS) devices have flow diversion effects (8). We treated unruptured large and giant partially thrombosed vertebral artery aneurysms by overlap stenting using multiple LVIS stents, because the use of FD for posterior circulation aneurysms is unauthorized in our country, and we focused on the morphological change of the stented artery. Also, CFD analysis was performed for understanding hemodynamic changes after stent replacement.

## Materials and Methods

### Patients

Between October 2017 and April 2019, four patients with large and giant partially thrombosed vertebral artery aneurysms were treated by overlap stenting using LVIS stents (MicroVention, Aliso Viejo, CA, USA). Their clinical information is summarized in Table 1. For the flow diversion effect, multiple LVIS stents were placed in the parent artery of the aneurysms. No coils were used in the treatment of any of the aneurysms described in this study. After treatment, patient follow-up included conventional digital subtraction angiography (DSA) and magnetic resonance imaging (MRI). We assessed both angiographic and clinical outcomes. The O'Kelly-Marotta (OKM) grading scale was used to assess the degree of angiographic filling and contrast stasis in the aneurysms.

**Table 1:** Clinical information.

**Case**	**Age**	**Dome size (mm)**	**Symptoms**	**Number of** **LVIS stent**	**Morphological change** **of parent artery**	**Angiographic outcome**	**Clinical outcome**
1	60–65	18	Dizziness	2	No	Complete occlusion at 2 years	Improved
2	70–75	23	Left hemiparesis, dysphasia	2	No	Complete occlusion at 2 weeks	Improved
3	50–55	38	Left hemiparesis	2	No	Almost complete occlusion at 6 months	Partially improved
4	50–55	35	Vertigo, left hemifacial spasm	5	Yes	Worsened	Worsened

*LVIS, low-profile visualized intraluminal support*.

For the treatment, antiplatelet therapy was administrated with 100 mg of aspirin and 75 mg of clopidogrel per day for 7 days before the procedure. After the procedure, the dual antiplatelet therapy was continued for at least 6 months, and clopidogrel was continued for 1–2 years.

### CFD Modeling and Analysis of Hemodynamic Parameters

**Figure 1:**
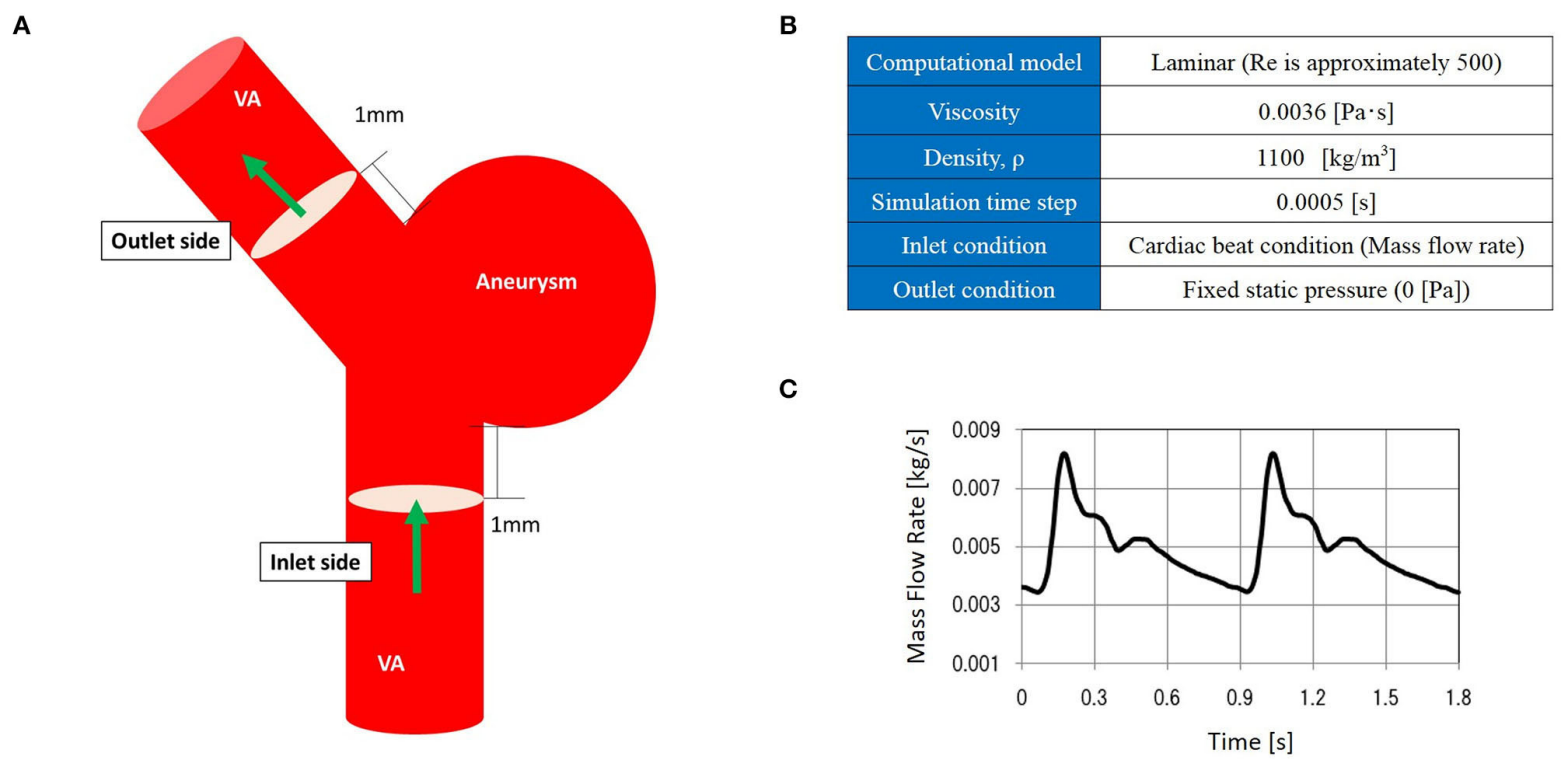
The computational fluid dynamics (CFD) model. CFD analysis domain **(A)**, CFD analysis condition **(B)**, and inlet mass flow rate **(C)**.

CFD analysis was performed to evaluate hemodynamics at the treatment site. Aneurysm and parent vessel geometries were extracted from DSA images via manual cropping and image thresholding. This information was subsequently converted to a triangulated surface using Amira Software (FEI Company, Hillsboro, OR, USA). A commercial software package (ANSYS ICEM CFD 18.21, ANSYS Inc., Canonsburg, PA, USA) was used to generate an unstructured computational volumetric mesh. This mesh mainly comprised tetrahedrons along with several prism element layers near the wall surface to increase the analytic precision of the boundary layer. The numbers of grid elements were ~1,500,000. After assuming a pulsatile laminar flow, pressure of 0 (Pa) at the outlet, and rigid blood vessel walls with non-slip conditions, the blood flow along the computational mesh was simulated using Navier–Stokes equations. The analysis domain encompassed the whole aneurysm dome from the aneurysm inlet side to the outlet side. Figure 1 shows additional details regarding the CFD modeling.

We investigated several hemodynamic parameters, focusing on pressure-related parameters. As previously described (9), the pressure difference (Pd) was defined as the degree of pressure elevation at the aneurysm wall. It is calculated by subtracting the average pressure (Pave), the mean pressure value in this domain, from the pressure of the target area (P). To normalize this value, it was divided by the dynamic pressure at the side of the aneurysm inlet.

(1) $$\begin{array}{l} Pressure\;difference=\frac{P-Pave}{\frac{1}{2}\rho Vin^{2}} \end{array}$$

**P: pressure [Pa], Pave: average pressure [Pa]**

**ρ: 1,100 [kg/m**^**3**^**], Vin: mean velocity of the aneurysm inlet [m/s]**

We also assessed wall shear stress (WSS) in the aneurysm dome, defined as the frictional force of blood flow along the aneurysm wall.

## Results

**Figure 2:**
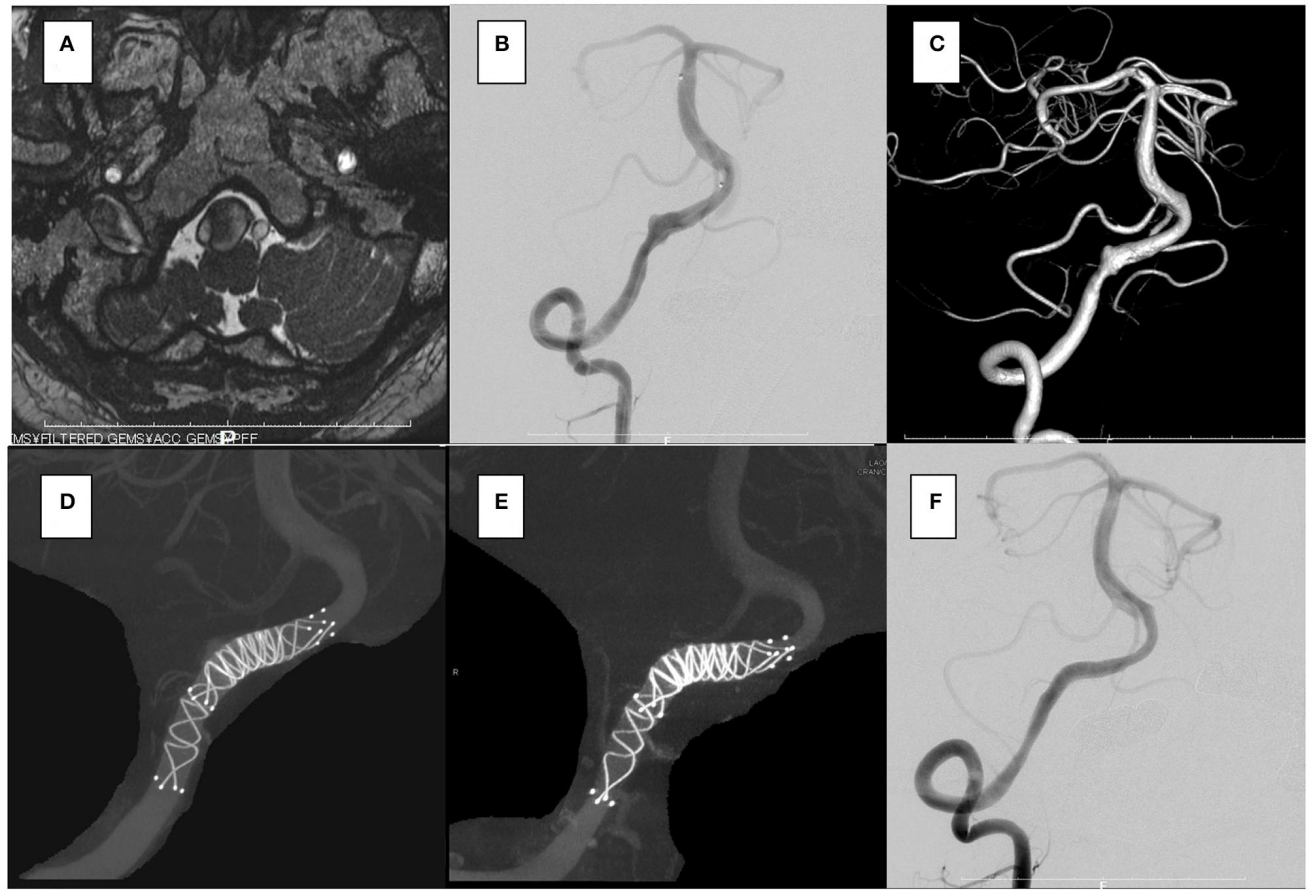
In the case 1, the patient presented with dizziness. The brain stem was compressed by a large partially thrombosed vertebral artery aneurysm. **(A–C)** Overlapping stents with double low-profile visualized intraluminal support (LVIS) were placed (two of 4.5 × 32 mm). **(D)** After 2 years, the follow-up angiography shows that the aneurysm was closed off **(E,F)**, and his symptoms were resolved.

**Figure 3:**
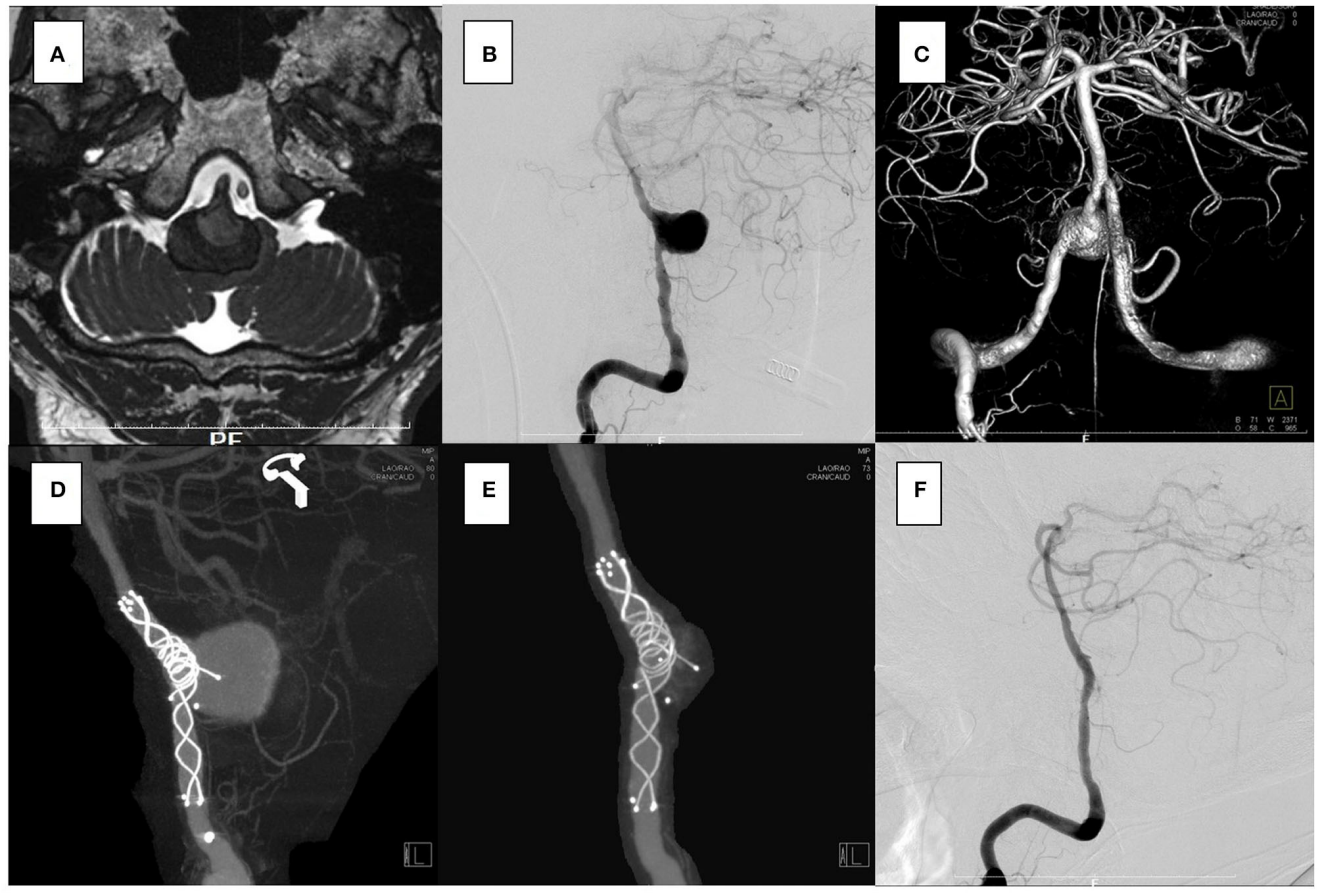
In the case 2, the patient presented with left hemiparesis and dysphasia. His brain stem was compressed by a large partially thrombosed vertebral artery aneurysm. **(A–C)** Overlapping stents with double low-profile visualized intraluminal support (LVIS) were placed (3.5 × 22 mm, 4.5 × 32 mm). The first stent was shortened when a micro catheter was advanced to the distal end of the vertebral artery, but it covered the aneurysm orifice. **(D)** After 2 weeks, the follow-up angiography showed that the aneurysm was closed off. **(E,F)** No recanalization has been seen for 2 years.

**Figure 4:**
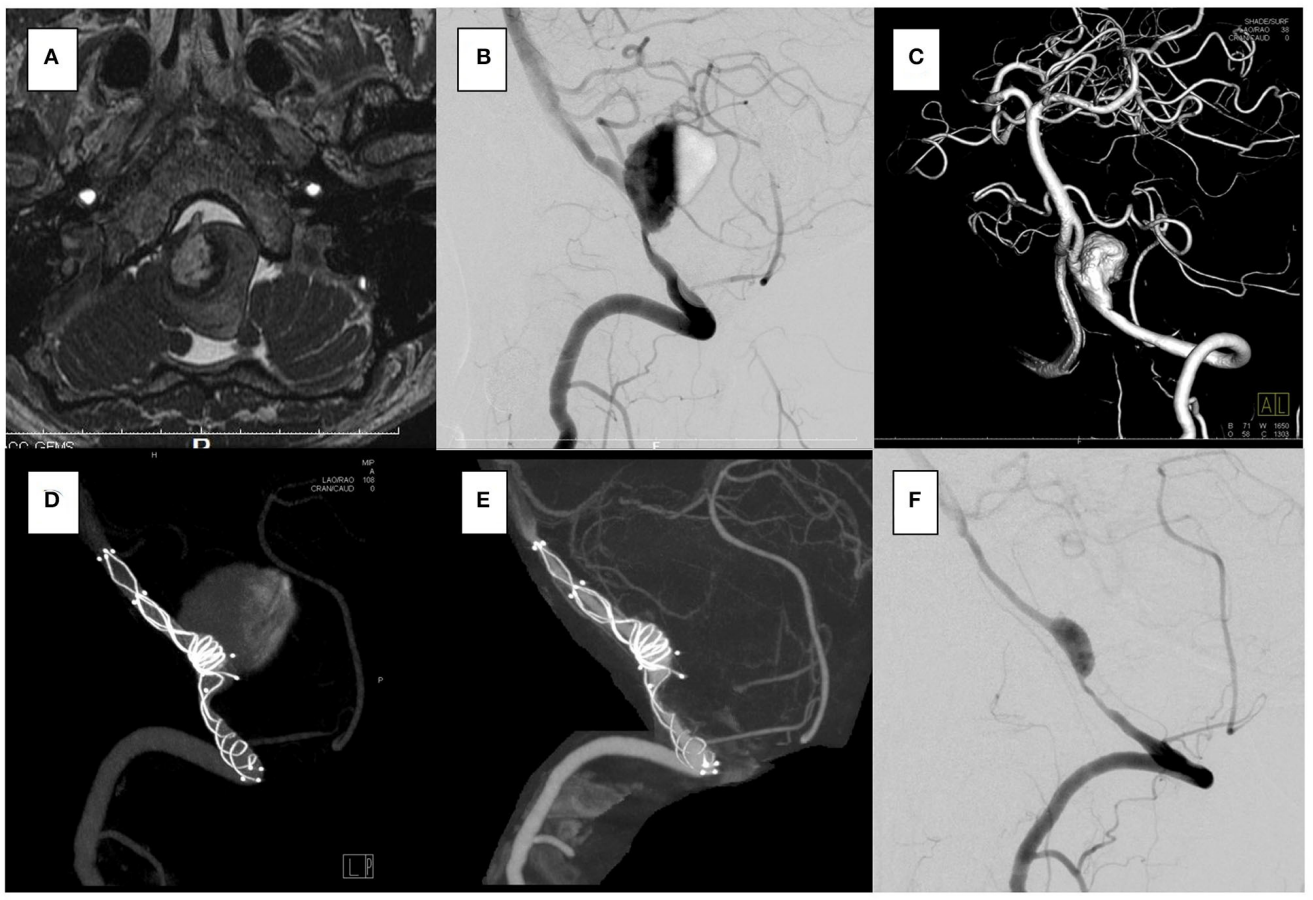
In the case 3, the patient presented with left hemiparesis. The brain stem was compressed by a giant partially thrombosed vertebral artery aneurysm. **(A–C)** Overlapping stents with double low-profile visualized intraluminal support (LVIS) were placed (4.0 × 22 mm, 4.5 × 32 mm). The first stent was shortened when a micro catheter was advanced to the distal end of the vertebral artery, but it covered the aneurysm orifice. **(D)** After 6 months, the follow-up angiography showed that the aneurysm was almost closed off. **(E,F)** No recanalization was seen for 1 year.

**Figure 5:**
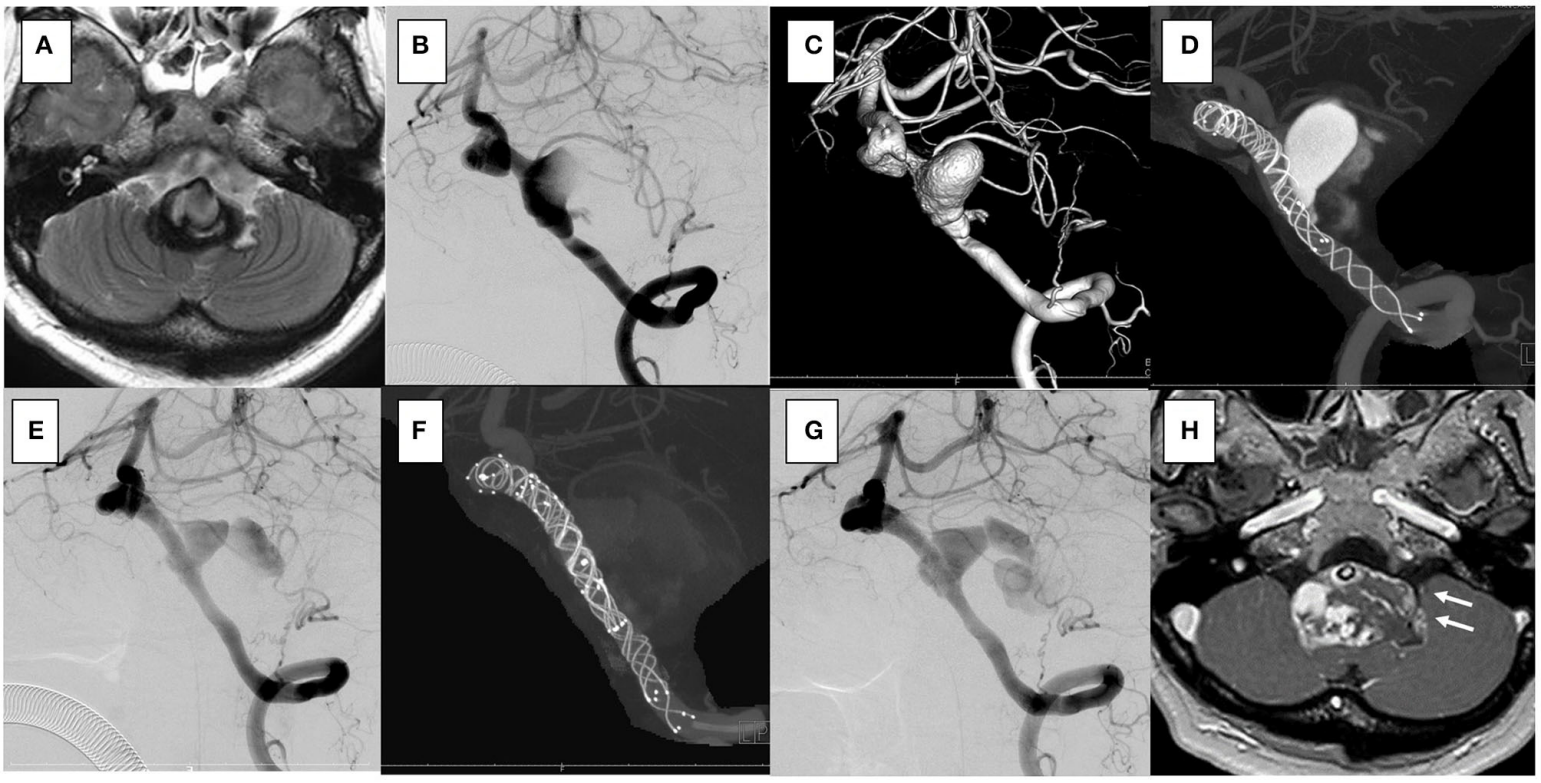
In the case 4, the patient presented with vertigo and left hemifacial spasm. The brain stem was compressed by a giant partially thrombosed vertebral artery aneurysm. **(A–C)** Overlapping stents with triple low-profile visualized intraluminal support (LVIS) were placed (three of 4.5 × 32 mm), and stagnation of flow occurred soon after treatment. Her symptoms were resolved. **(D)** After 1 year, the follow-up angiography showed recanalization. **(E)** Additional stents with double LVIS were placed (4.5 × 18 mm, 4.5 × 23 mm). **(F)** However, it was uncontrollable and further recanalization was seen in the 6-month follow-up angiography **(G)**; furthermore, progressive aneurysmal enlargement occurred and aneurysm wall enhancement indicating the vasa vasorum was observed [**(H)**; white arrow]. Clinically, she presented vertigo and hemifacial spasm again, and slight hoarseness was apparent.

**Figure 6:**
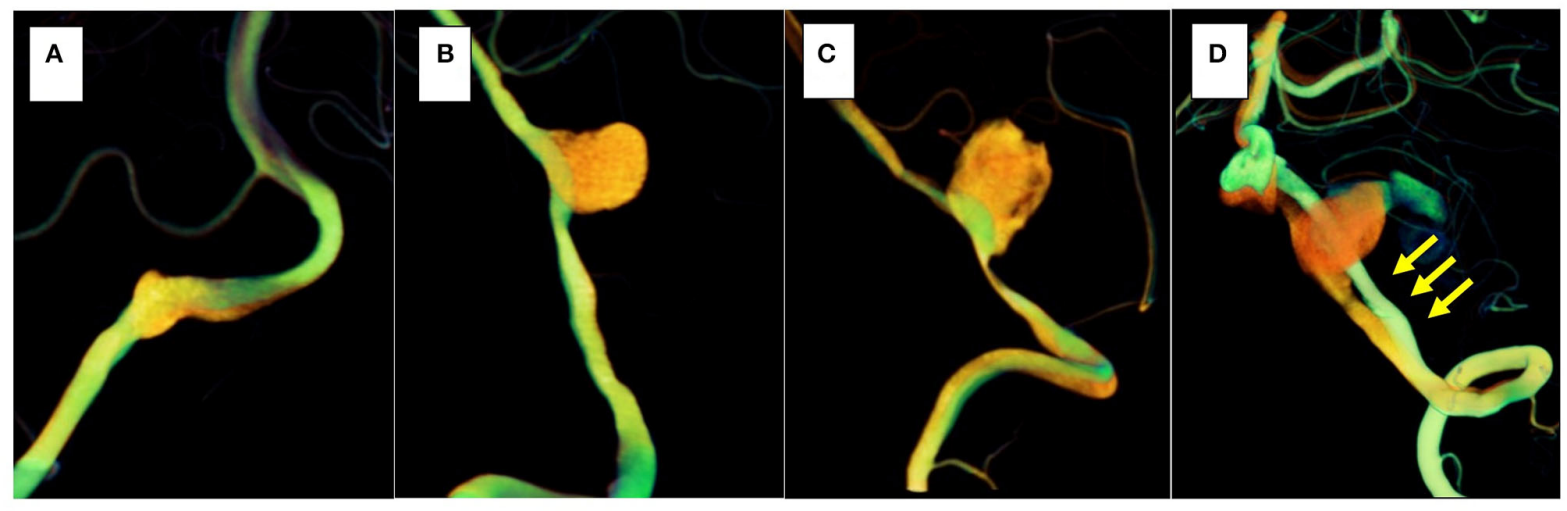
The orange vessel is pre-treatment, and the green vessel is post-treatment. In three cases (cases 1–3), morphological changes in the stented parent artery were not seen **(A–C)**; however, in the recurrent case (case 4), the parent artery straightened after stent placement (yellow arrows) **(D)**.

**Figure 7:**
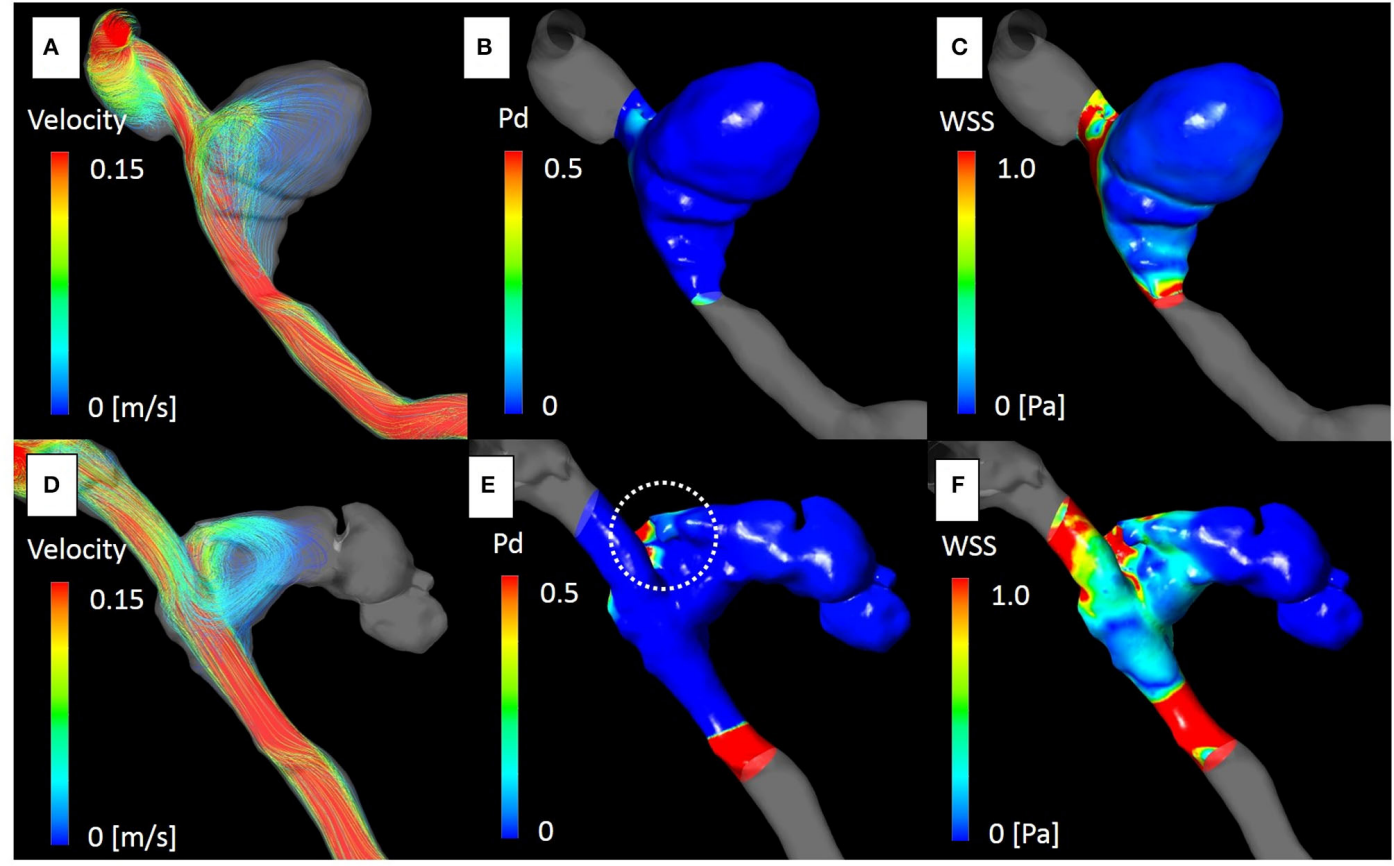
The computational fluid dynamics study of case 4. Streamline, pressure difference (Pd) and wall shear stress (WSS) at the peak systolic phase before [**(A–C)**, respectively], and after treatment [**(D–F)**, respectively]. Blood flow into the aneurysm dome was altered after placement of the overlapping stents due to morphological changes in the parent artery **(D)**. Pd around the aneurysm neck was significantly elevated [**(E)**; white dot circle]. The maximum Pd identical to the impingement zone of the aneurysm dome was elevated from 0.08 to 1.01. WSS was generally elevated around the area **(F)**.

The aneurysm domes were occluded in two aneurysms and almost occluded in one aneurysm in the follow-up angiography (Figures 2–4). The clinical symptoms gradually resolved in the three cases. On the contrary, one giant vertebral thrombosed aneurysm was uncontrollable after overlap stenting with triple LVIS stents. Additional double LVIS stents were placed 12 months after the initial treatment; however, this worsened the aneurysm further by increasing inflow into the aneurysm dome and causing the enlargement of the thrombosed aneurysm (Figure 5). The morphological change of the parent artery was seen in the recurrent case after the initial treatment (Figure 6) and CFD analysis reveals that this change led to pressure elevation around the aneurysm neck. The highest Pd at the area increased from 0.08 to 1.17, while WSS generally remained elevated (Figure 7).

## Discussion

Recently, FDs have been widely used for the treatment of cerebral aneurysms (10, 11). The effectiveness of FDs for large and giant thrombosed aneurysms has been reported, with curative thrombosis of the aneurysm sac caused by reducing flow, without the use of coils (12). Posterior circulation thrombosed aneurysms are particularly difficult to treat because of the high rate of recanalization and compression of the brainstem, which coil embolization has the potential risk of worsening (13, 14). The effect of flow diversion is, at least theoretically, useful for the treatment of such aneurysms. In our study, overlap stenting with braided stents was used because FD is unauthorized for use in posterior circulation aneurysms in our country. Flow reduction was achieved in three out of four aneurysms by overlap stenting. It has been reported that multiple overlapping LVIS stents have a similar flow diversion effect as that of FD (6, 8). Metal coverage of the aneurysm orifice is one of the important factors causing a flow diversion effect. The porosity of the braided stents also contributes to decreased flow into the aneurysms (15, 16). We especially focused on the morphological changes in the stented parent artery, which previous studies have reported as contributing to a flow diversion effect (1–4). Ishii et al. analyzed unruptured large aneurysms with and without a stent and reported that the neck-bridging stent prevents recanalization caused by the straightening effect of the parent artery, most likely caused by significant angular change (1). Additionally, Larrabide et al. studied aneurysms in the internal carotid artery using CFD before and after FD treatment (4). They reported that successful blood flow reduction to the site of the aneurysm was related to aneurysm position and orientation with respect to the parent vessel, in addition to the morphology of the aneurysm. These studies reported that flow diversion after morphological changes of the stented parent artery can result in a positive outcome. Interestingly, in case 4, the morphological changes of the parent artery caused by multiple overlapping stents led to increased pressure and WSS in the aneurysm. Additional stents were not effective, even though the mesh porosity was theoretically much lower than that of FD. These results highlight that flow diversion effects with morphological changes to the parent artery can worsen due to blood flow into an aneurysm sac. Previous studies have reported adverse events associated with aneurysm rupture after treatment with FD, and the researchers speculated that flow diversion causes inflow jet and pressure elevation in the aneurysm sac (17–20). Some studies reported that failure to prevent increases in intra-aneurysmal pressure can occur because of parent artery configuration and curvature (20, 21). In addition, the elevation of WSS on the neck wall caused by altered blood flow is thought to increase the risk of recanalization (22, 23). Although recent engineering studies have analyzed the hemodynamic changes after stenting, it remains unclear how and to what extent the parent artery changes after stent placement (24, 25).

From a different perspective, the vasa vasorum is thought to play an important role in wall remodeling in the progressive growth of thrombosed large and giant aneurysms, and the image of aneurysm wall enhancement on MRI indicates inflammatory etiology including the vasa vasorum, which suggests malignant behavior of the aneurysm growth (26, 27). This finding was observed in the recurrent case even after retreatment and suggested intractable aneurysm due to worsening inflammation (Figure 5H).

Our study is the first to report a flow diversion effect by overlapping neck-bridge stents without coils for large and giant partially thrombosed vertebral artery aneurysms; however, CFD analysis of a recurrent case revealed that negative outcomes were related to changes in blood flow dynamics due to morphological changes in the stented parent artery.

There are some limitations to this study. First, multiple stents were not simulated because it is not possible to acquire the morphological data of multiple LVIS stents, and this may influence the validity of our results. Second, the number of cases for analysis was small. Additional studies describing larger series are required. Third, the follow-up period may have been inadequate to determine whether the treatment was curative. Fourth, vasa vasorum is one of the important factors that causes the enlargement of thrombotic aneurysms. However, the other three cases of vasa vasorum were not available as contrast enhanced MRI was not performed routinely at our institution, and therefore, these could not be included for a comparison in this series.

## Conclusion

Overlapping stents can be used to treat large and giant thrombosed vertebral artery aneurysms with a flow diversion effect. However, morphological changes of the stented parent artery may increase intra-aneurysmal flow causing a negative outcome, and careful attention is needed in this regard.

## Data Availability Statement

The original contributions presented in the study are included in the article/supplementary material, further inquiries can be directed to the corresponding author/s.

## Ethics Statement

The studies involving human participants were reviewed and approved by The ethics committee of Niigata University Medical and Dental Hospital (approval number: 2020-0027). Written informed consent for participation was not required for this study in accordance with the national legislation and the institutional requirements.

## Author Contributions

TS designed the study, acquired and analyzed the data, and drafted the article. All authors critically revised the article for important intellectual content and approved the final version for publication.

## Conflict of Interest

The authors declare that the research was conducted in the absence of any commercial or financial relationships that could be construed as a potential conflict of interest.
